# Solitary pulmonary metastasis from prostate cancer with neuroendocrine differentiation: a case report and review of relevant cases from the literature

**DOI:** 10.1186/s12957-015-0598-2

**Published:** 2015-05-07

**Authors:** Toshiya Maebayashi, Katsumi Abe, Takuya Aizawa, Masakuni Sakaguchi, Naoya Ishibash, Shoko Fukushima, Taku Honma, Yoshiaki Kusumi, Tsuyoshi Matsui, Nozomu Kawata

**Affiliations:** Department of Radiology, Nihon University School of Medicine, 30-1 Oyaguchi kami-cho, Itabashi-ku, Tokyo 173-8610 Japan; Department of Pathology, Nihon University School of Medicine, 30-1 Oyaguchi kami-cho, Itabashi-ku, Tokyo 173-8610 Japan; Department of Urology, Nihon University School of Medicine, 30-1 Oyaguchi kami-cho, Itabashi-ku, Tokyo 173-8610 Japan

**Keywords:** Solitary lung metastasis, Prostate cancer, Neuroendocrine differentiation

## Abstract

**Background:**

Solitary lung metastasis from prostate cancer is rare. There are few reports of such cases with neuroendocrine differentiation.

**Case presentation:**

A 50-year-old man presented to our hospital with a chief complaint of dysuria. Histological examination revealed prostate cancer, which was classified as cT4 N0 M0, stage IV adenocarcinoma. Since the patient was at high risk, endocrine and radiation therapies were started. One year after starting radiation therapy, the patient developed bloody sputum. Chest radiography revealed a nodular shadow in his left lung (S5). Although 18-fluoro-2-deoxyglucose positron emission tomography revealed abnormal accumulation in the lesion, the cytological diagnosis was class IIIa, which did not yield a definitive diagnosis. Given that prostate specific antigen (PSA) was not elevated, a primary lung tumor was suspected, and thoracoscopic segmental resection of the lung was performed with lymph node dissection. The final pathological diagnosis was solitary lung metastasis from prostate cancer with neuroendocrine differentiation and mediastinal lymph node metastasis. The specimen showed a mixed pattern of conventional prostatic and neuroendocrine carcinomas.

**Conclusion:**

We herein report a case with neuroendocrine differentiation (NED), along with a review of the relevant literature, including histopathological findings. According to previous case reports, some patients with solitary lung metastasis from prostate cancer achieved relatively good long-term survival. We consider establishing the correct diagnosis and implementing an appropriate treatment plan to be essential in prostate cancer patients with oligometastases that have the potential to be neuroendocrine (NE) tumors.

## Background

Solitary lung metastasis from prostate cancer is rare. To date, only 22 cases have been reported (including our present patient). There are few reports of such cases with neuroendocrine differentiation (NED). NED in prostate carcinoma occurs in the form of a small cell carcinoma, carcinoid or carcinoid-like tumor, and as focal NED in conventional prostatic adenocarcinoma [1]. Neuroendocrine (NE) cells exist in the normal prostate and are considered to be involved in the differentiation and proliferation of prostatic cells through various growth factors, without mediation by androgen receptors (AR) [1]. In addition, NE tumors can be classified according to the conditions at the time of detection into three types as follows: pure NE prostate carcinoma at the time of initial examination, mixed pattern of conventional and NE prostatic carcinomas at the time of initial examination, and NE carcinoma (NEC) developing during endocrine therapy [2]. We herein report a case with NED, along with a review of the literature describing similar cases, including histopathological findings.

## Case presentation

**Figure 1:**
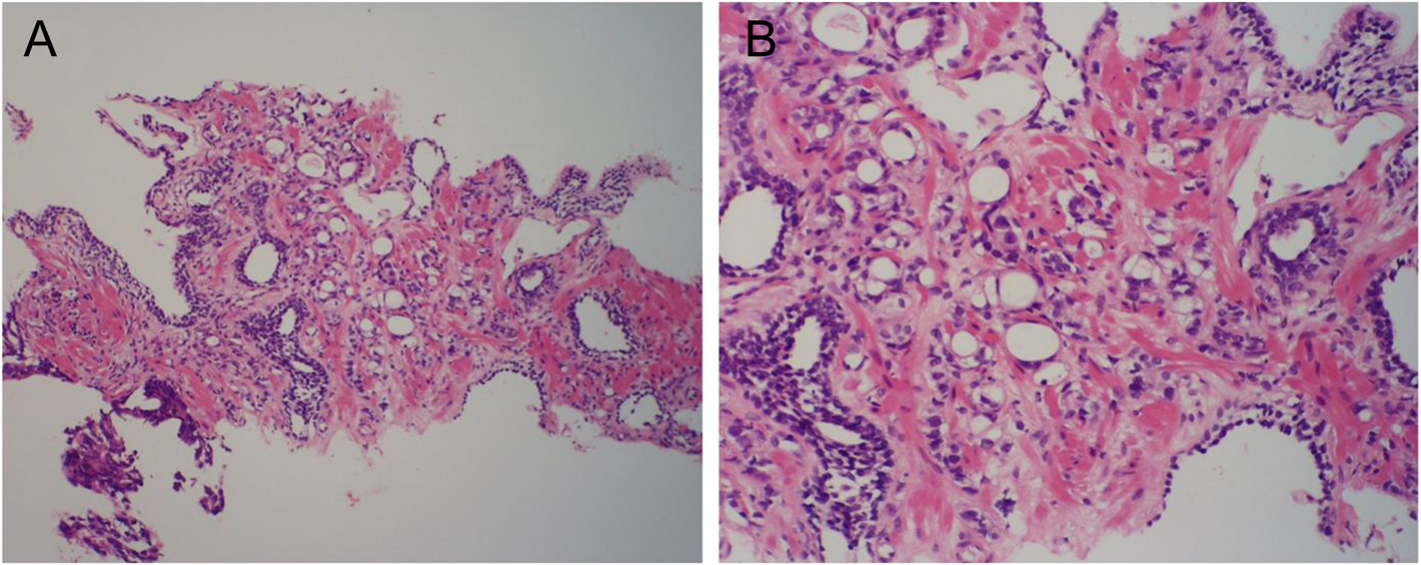
Histopathology of the prostatic needle biopsy at the time of initial examination. Conventional prostate adenocarcinoma with tubular and cord-like structures, corresponding to Gleason score 4 + 5 = 9, is seen in the specimen (**A**, ×100; **B**, ×200).

A 50-year-old man presented with a chief complaint of dysuria. At the Outpatient Ward of our hospital, he was clinically suspected of having prostatic cancer based on the results of various clinical examinations including a high prostate-specific antigen (PSA, 61.77 ng/ml) value. A prostatic needle biopsy was thus performed. Histopathologically, the biopsy specimens showed adenocarcinoma forming medium to small glandular and cord-like structures. The patient was thus diagnosed as having a poorly differentiated conventional prostate adenocarcinoma with a Gleason score of 4 + 5 = 9 (Figure 1A, B). According to the imaging and biopsy findings, the tumor was classified as cT4 N0 M0, stage IV.

Cystoscopic examination revealed an erythematous velvety appearance of the bladder mucosa, indicating that the tumor was attributable to direct infiltration of prostate carcinoma, consistent with the classification cT4 N0 M0, stage IV. The patient was considered to be at a high risk for the spread of prostatic cancer, and endocrine therapy was started using goserelin acetate and bicalutamide. One month after starting this endocrine therapy, the patient underwent local radiation treatment at a dose of 66 Gy. After these endocrine and radiation therapies, his PSA levels gradually decreased but remained above the upper limit of the reference range.

**Figure 2:**
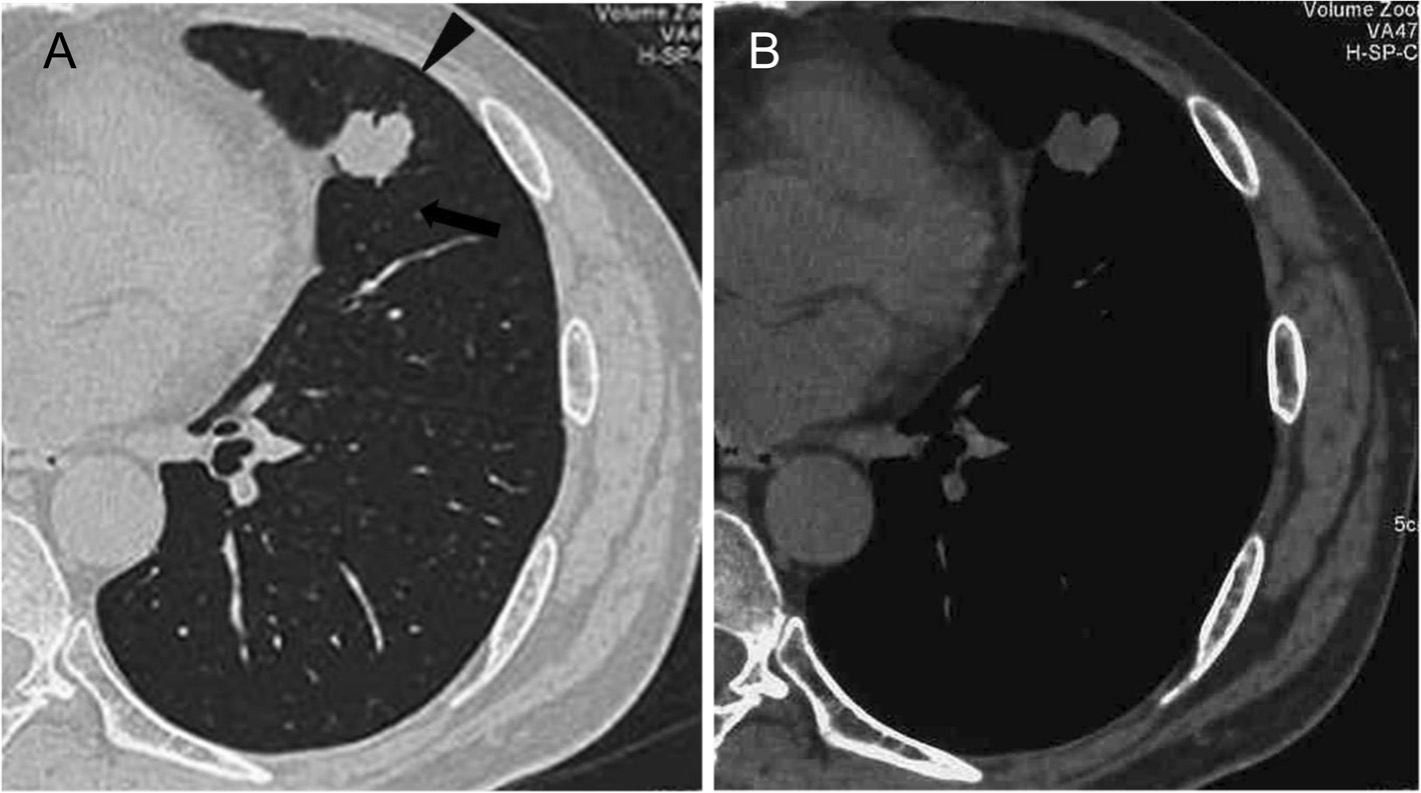
Plain computed tomographic image (lung window) showing the nodes with spicula (*arrowhead*), notch (*arrow*), and pleural indentation in the lingual segment of the left lung. There is no evidence of calcification. **(A)** Lung window and **(B)** mediastinal window.

**Figure 3:**
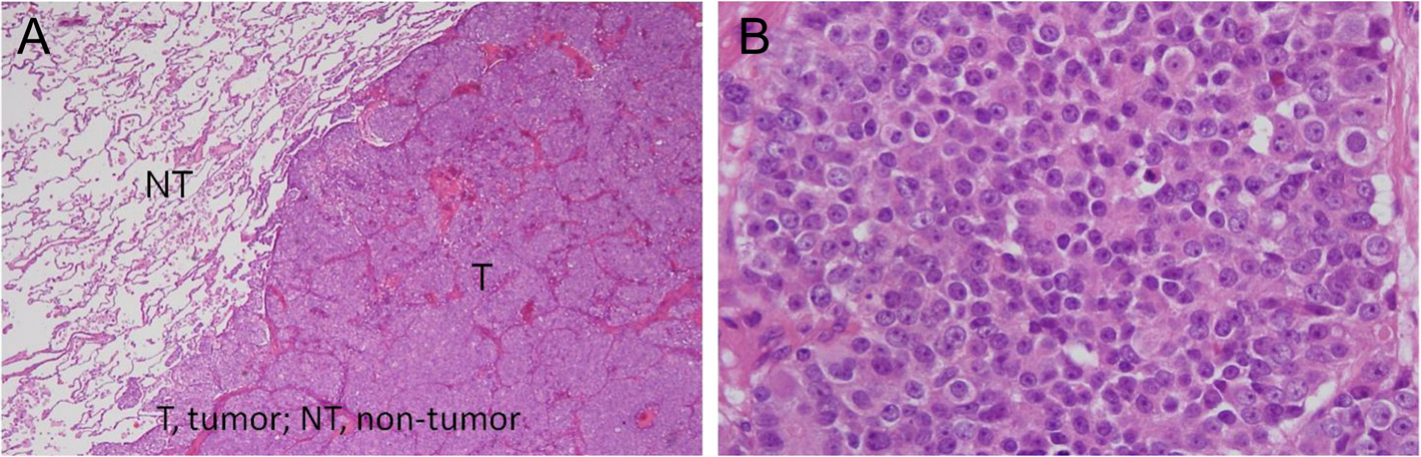
Histopathology of the lung tumor. Pathological tissues stained with hematoxylin-eosin. The lung tumor (T) is well-demarcated from the non-neoplastic lung tissue (NT) (**A**, ×20). The tumor is composed of eosinophilic tumor cells with swollen ovale nuclei and prominent nucleoli showing a sheet-like pattern of growth (**B**, ×200).

**Figure 4:**
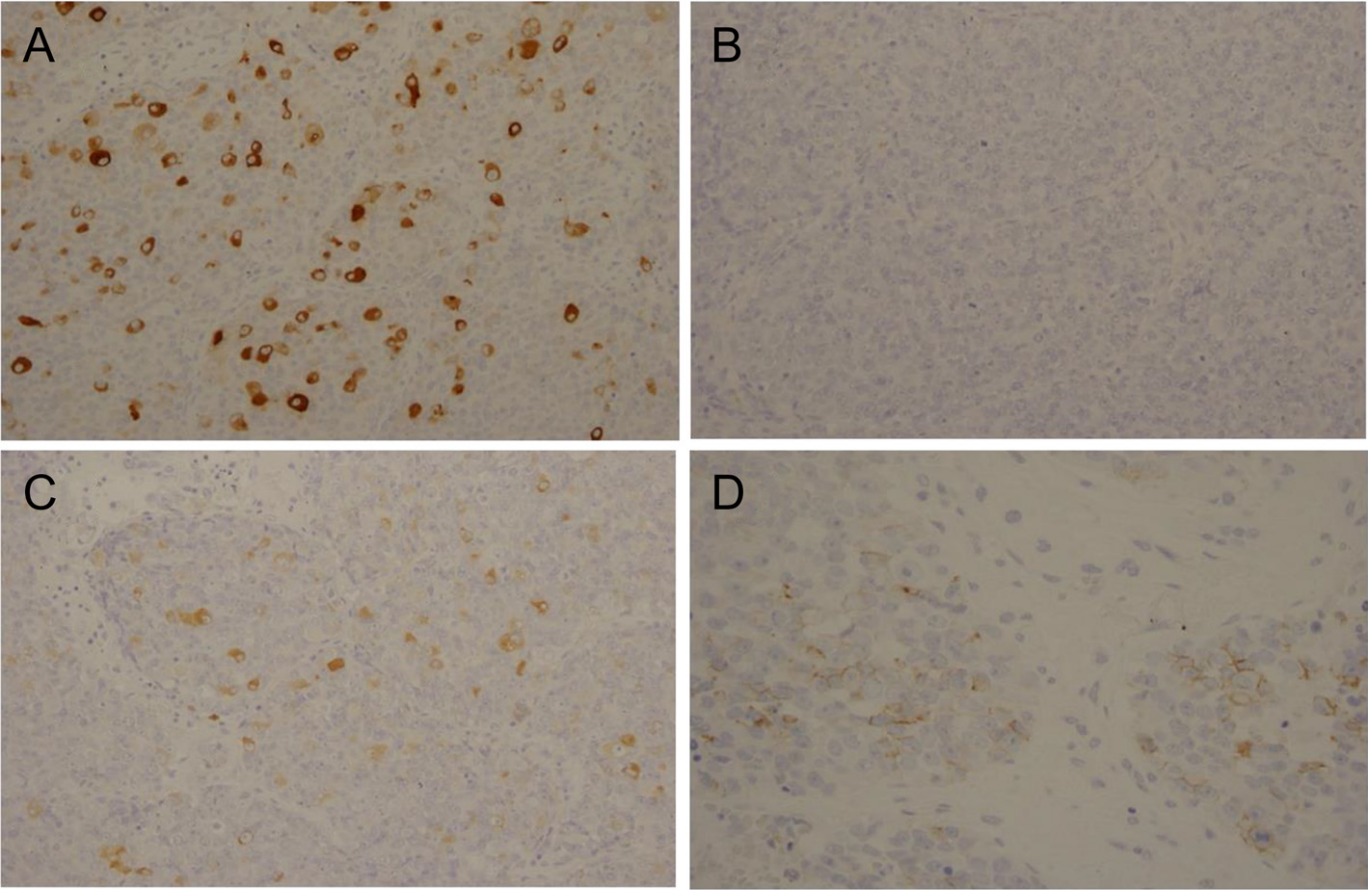
Immunohistochemistry of the lung tumor. The lung tumor was positive for PSA **(A)**, synaptophysin **(C),** and CD56 **(D)**, but negative for surfactant apoprotein **(B)**. All images are ×200.

One year after starting radiation therapy, the patient developed bloody sputum. A computed tomographic scan showed 3-cm nodes, with spicula, notch, and pleural indentation, in the lingual segment of the left lung (Figure 2A, B). We then applied 18-fluoro-2-deoxyglucose positron emission tomography which revealed selective accumulation in the nodes, strongly suggesting a malignant tumor. Given that the PSA level was not markedly elevated, the patient was suspected to have a primary lung tumor and underwent thoracoscopic segmental resection of this portion of the lung with lymph node dissection. Histopathological examination of the resected lung revealed a neoplastic lesion. The tumor was highly cellular and composed of eosinophilic tumor cells with swollen ovale nuclei and prominent nucleoli. The tumor cells exhibited a sheet-like pattern, and a partly glandular pattern, of growth (Figure 3A, B). Furthermore, the tumor cells proliferated along the alveolar septae, replacing the alveolar lining epithelial cells. There was no evidence of pleural infiltration of the tumor cells. Immunohistochemical analysis results were positive for not only PSA (Figure 4A) but also chromogranin, synaptophysin (Figure 4C), and CD56/CD57 (Figure 4D), suggesting NED. None of the tumor cells was immunopositive for surfactant apoprotein (Figure 4B) or CK20/CK7. Therefore, although this tumor with NED proliferated along the alveolar septae and showed a relatively infrequent glandular pattern of growth, we considered the lung tumor to be a solitary pulmonary metastasis from prostate adenocarcinoma.

The patient subsequently received chemotherapy. In this case, relatively high doses of paclitaxel (120 mg/m^2^) and carboplatin (dosed to a target area under the concentration-time curve [AUC] of 6.0) were administered every 4 weeks for 2 cycles. After adjuvant chemotherapy, the patient was treated with irradiation of the entire mediastinal field at a dose of 60 Gy with a boost field of 10Gy. Thereafter, the patient developed systemic metastases and received both chemotherapy and radiation therapy for multiple lesions. He survived for approximately 30 months before dying of systemic metastases.

### Discussion

**Figure 5:**
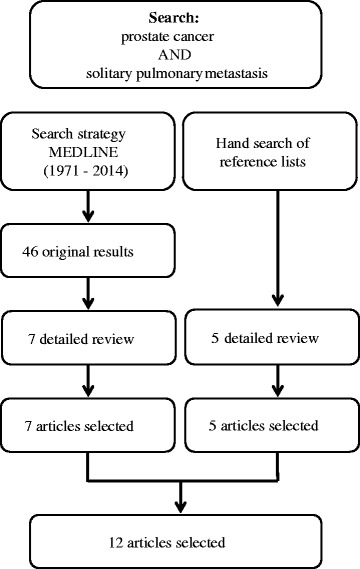
Flow chart of search strategy. Twelve articles were identified.

The incidence of lung metastasis from prostate cancer reported ranges from 5% to 27% [3]. Fabozzi *et al*. reported the incidence of solitary lung metastasis from prostate cancer to be only 0.86% [3]. Our search of PubMed identified 46 articles with prostate cancer and solitary pulmonary metastasis. In total, seven publications [4-10] on cases similar to ours were identified, and all full texts were retrieved. In total, 12 articles describing 22 cases were identified, but we looked for 5 articles [3,11-14] from references of past reports (Figure 5). To date, only 23 cases (including our present patient) with solitary lung metastasis from prostate cancers have been reported (Table 1) [3-14]. The present patient is the first, to our knowledge, with solitary lung metastasis from prostate cancer with NED.

**Table 1:** **Clinical data of patients with solitary lung metastasis of prostatic cancer**

**Author**	**Year**	**Age (years)**	**Initial characteristic**	**Initial treatment**	**Adjuvant/salvage treatment**	**Lung meta. characteristics**	**Solitary lung meta. treatment**	**Pathology**	**Outcome**
Present case		50	PSA 61.7, cT4, GS4 + 5	Neoadjuvant endocrine therapy radiation therapy	None	Left S5, 3 cm	Lobectomy and mediastinal LND	PSAP (+), chromogranin (+), synaptophysin (+), CD56/CD57 (+), surfactant apoprotein (−), CK20/CK7 (−), mediastinal LN meta	Died 2.5 years after metastases detected
Pepe *et al*. [6]	2012	75	PSA 8.3, pT3a, GS4 + 3, mixed acinar and ductal prostate ca	Radical prostatectomy	None	Left S6, 2 cm	Metastatectomy	PSAP (+), TTF-1 (−)	Undetectable
Sakai *et al*. [7]	2010	74	Unknown	Neoadjuvant endocrine therapy radiation therapy	Neoadjuvant endocrine therapy	Left S8, 2.2 cm	Wedge resection	PSAP (+), P504 (+), pleural dissemination	Undetectable
Goto *et al*. [9]	2010	73	PSA 14.37, GS4 + 5, pT4, P/D adenoca with sarcomatoid carcinoma component	Nneoadjuvant endocrine therapy. pelvic evisceration	None	Left S3, 2 cm	Wedge resection	PSAP (−), cytokeratin A/E1/3 (+), cytokeratin7 (−), cytokeratin20 (−), vimentin (−), undifferentiated carcinoma	Undetectable
Boyer *et al*. [13]	2009	65	PSA 3, GS3 + 3, pT2	Radical prostatectomy	None	Left upper lobe, 2.8 cm	Surgical resection	Unknown	Undetectable
Khandani *et al*. [12]	2009	78	Prostate cancer only	Radiation therapy	None	Left S10, 5 cm	Lobectomy and mediastinal LND	PSAP (−)	Undetectable
Pruthi *et al*. [10]	2007	72	PSA 4.1, GS3 + 3, pT2b	radical prostatectomy	salvage radiation therapy	left S8, 2 cm	endocrine therapy and surgical resection	PSAP (+), GS 8, subcarinal LN meta	3-year disease free follow up
Chao *et al*. [4]	2004	68	PSA 6.7, GS4 + 5, pT2a	Radical prostatectomy	None	Left lower lobe, 1.2 cm	Wedge resection	PSAP (+), W-M/D adenoca	12-year disease free follow up
Hofland *et al*. [8]	2000	49	GS 4 + 5, pT3c, P/D adenoca	Radical prostatectomy	Salvage radiation therapy, endocrine therapy and orchiectomy	Left lower lobe only	Lobectomy	PSAP (+), P/D adenoca	Metastases, undetectable
Smith *et al*. [5]	1999	70	GS4 + 5, pT2	Radical prostatectomy	None	Right S7, 2 cm	Surgical resection	PSAP (+), P/D adenoca	Undetectable
Rockey *et al*. [14]	1990	83	Low grade prostate cancer only	Radiation therapy	None	Left lower lobe only	Orchiectomy	PSAP (+), meta. Only	Undetectable
Fabozzi *et al*. [3]	1995	11 cases: details unknown							
Varkarakis *et al*. [11]	1974	One case, details unknown							

‘+’, Positive; PSA: prostate-specific antigen; NEC: neuroendocrine carcinoma; NET: neuroendocrine tumor; NENs: neuroendocrine neoplasms; GS: Gleason score; PSAP: prostate-specific antigen phosphatase; CD56/57: Neural cell adhesion molecule/Leu 7; adenoca: adenocarcinoma; LND: lymph node dissection.

In the early years of the study of this tumor type, endocrine tumors were designated differently depending on the site of origin. In 1907, Oberndorfer proposed the name ‘carcinoid’ for endocrine tumors that develop in the gastrointestinal tract [15]. Then, the idea that these tumors arise from endocrine cells and disperse throughout the body gained acceptance and led to a shared recognition of carcinoid and other endocrine tumors. In 2004, the World Health Organization (WHO) proposed including the ambiguous term carcinoid in the category of endocrine tumors. According to the 2004 WHO classification, NE tumors (NET) in the lung were classified into small cell carcinoma, large cell neuroendocrine carcinoma, typical carcinoid tumor, and atypical carcinoid tumor [16]. In this classification, the former two tumor types (small cell and large cell neuroendocrine carcinomas (NECs)) were defined as poorly differentiated NEC, whereas the latter two (typical and atypical carcinoid tumors) were deemed endocrine tumors arising in the gastrointestinal tract and defined as well-differentiated NET.

Furthermore, in 2010, the WHO proposed giving pancreatic and gastrointestinal tumors with an endocrine phenotype the name neuroendocrine neoplasms (NENs) [17]. In this classification system, NENs are divided into well-differentiated NETs and poorly differentiated NECs, and the NETs are further subdivided according to the number of mitoses and the percentage of ki67 staining, both of which are indices of cellular proliferation.

Since the 1990s, NE prostate cancers have been classified into small cell carcinoma, carcinoid or carcinoid-like tumor, and prostate adenocarcinoma with NED [1]. NE cells exist in the normal prostate and secrete growth factors and cytokines, including serotonin, calcitonin, secretin, and other peptide hormones. In addition, NE cells are considered to be involved in the differentiation and proliferation of prostatic cells through growth factors, and these processes are not mediated by ARs [1]. However, there are various hypotheses explaining their possible histogenesis, including the following: 1) the tissues are derived from preexisting NE cells in the prostate gland, 2) the tissues develop through differentiation of adenocarcinoma, and 3) the tissues arise from multi-potential stem cells [18]. NET in prostate cancer is rare, with reported prevalences of 0.2% to 1% [2,19]. NETs can be classified according to the conditions at the time of detection as pure NE prostate carcinoma at the time of initial examination, mixed pattern of conventional and NE prostatic carcinomas at the time of initial examination, or NEC that develop during endocrine therapy. A Japanese study found the incidence rates of these three conditions to be 35%, 18%, and 47%, respectively [20]. Japan has a higher incidence of NED in prostate cancer during endocrine therapy than other countries [2]. We speculate that, in the present case, NE cancer developed during endocrine therapy for conventional prostate adenocarcinoma.

In this patient, the lung mass was not definitively diagnosed preoperatively. The tumor was considered to be an adenocarcinoma replacing the alveolar lining cells based on an intraoperative rapid diagnosis, but we were unable to determine whether or not the tumor was a metastasis at this time. Imaging studies revealed spicula, notch, and pleural indentation findings, which also made differentiating between lung metastasis and a primary lung tumor difficult. One reason for the difficulty in differentiating between primary and metastatic lung tumors is that the replacement growth of alveolar lining epithelial cells is also observed in approximately 15% of metastatic lung tumors. Such a growth pattern occurs in many cancers, including colon, gallbladder, breast, gastric, prostate, thyroid, and kidney malignancies [21]. As a result, our present patient who was considered to have a primary lung cancer, underwent thoracoscopic segmental resection of the lung with lymph node dissection and then, finally, the lung mass was diagnosed as a metastatic adenocarcinoma with NED from the prostatic cancer.

Among the 23 case reports describing solitary lung metastasis (including the present patient), the treatment procedure was described for 11. Ten of these eleven patients underwent surgery, and most had good outcomes, but there were two the exceptions (case 6 and our present patient). In addition, the effectiveness of surgical treatment for solitary metastasis from prostate cancer has also been reported [4,22].

The present patient developed systemic metastases after surgery, necessitating chemotherapy and radiotherapy. This patient survived for approximately 30 months. NE cancer in the prostate gland has been regarded as having a poor prognosis, with reported median survival times of 9.8 months [19] and 13.1 months [23]. The relatively long survival time in the present case may be attributable to the pathological status of the disease; that is, the tumor demonstrated a mixed pattern consisting of conventional and NE prostatic carcinomas. The long-established protocol for patients with this condition is to administer treatment that includes chemotherapy for small cell lung cancer. Considering that the standard treatment of pulmonary small cell carcinoma has not changed since the 1990s [24], it may be difficult to achieve prognostic improvement, at least for now. We therefore consider immunostaining to be advisable at the time of biopsy for young patients with high-grade tumors in order to investigate the possible presence of NE tumors or NE components. Establishing the correct diagnosis and implementing the most appropriate treatment plan are important for reducing the impact of this disease.

## Conclusions

Solitary lung metastasis from prostate cancer is rare. We advocate that clinicians be aware of the possible presence of metastasis from prostate cancer when a patient has a solitary lung tumor histologically diagnosed along with NED or has a history of prostate cancer. According to the previously published case reports, some patients with solitary lung metastasis from prostate cancer achieved relatively good long-term survival. We consider establishing the correct diagnosis and implementing the most appropriate treatment plan to be essential in prostate cancer patients with oligometastases, if they have a NE tumor.

## Consent

Written informed consent was obtained from the patient for publication of this case report and any accompanying images. A copy of the written consent is available for review by the Editor-in-Chief of this journal.

## Abbreviations

NED: neuroendocrine differentiation
NE: neuroendocrine
AR: androgen receptors
WHO: World Health Organization
PSA: prostate-specific antigen
NEC: neuroendocrine carcinoma
NET: neuroendocrine tumor
NENs: neuroendocrine neoplasms
GS: gleason score
PSAP: prostate-specific antigen phosphatase
CD56/57: neural cell adhesion molecule/Leu 7
adenoca: adenocarcinoma
LN: lymph node
meta: metastasis
TTF-1: thyroid transcription factor-1
W-M/D: well to moderate differentiated
P/D: poorly differentiated
